# SOCIAL DETERMINANTS OF HOSPITAL READMISSION FOR OLDER VETERANS

**DOI:** 10.1093/geroni/igac059.723

**Published:** 2022-12-20

**Authors:** Portia Cornell, Christopher Halladay, Gina Chmelka, Caitlin Celardo, Robert Burke, Jennifer Silva, James Rudolph, Kali Thomas

**Affiliations:** Providence VA Medical Center / Brown University, Providence, Rhode Island, United States; Providence VA Medical Center, Providence, Rhode Island, United States; Veterans Health Administration, Washington, District of Columbia, United States; Veterans Health Administration, Washington, District of Columbia, United States; University of Pennsylvania Perelman School of Medicine, Philadelphia, Pennsylvania, United States; Veterans Health Administration, Washington, District of Columbia, United States; Veterans Health Administration, Providence, Rhode Island, United States; Brown University, Providence, Rhode Island, United States

## Abstract

The objective of this study was to estimate the effect of social risk on the likelihood of hospital readmission. Our study sample included 156,690 hospitalizations from 2016 - 2019 at one of 36 VA medical centers that participated in a national social-work staffing program. Using information from outpatient screenings, social workers’ assessments, and diagnosis codes, We identified Veterans’ social risks categorized into nine specific categories: intimate partner violence, financial need, housing instability, legal problems, social isolation, mental health, transportation, food insecurity, and functional need; and two general categories: nonspecific psychosocial and neighborhood deprivation. We estimated linear probability models of unplanned hospital readmission to a VA or a community hospital within 30 days of discharge, adjusted for demographics, clinical characteristics known to predict readmission (length of stay, primary diagnosis, admission from emergency department, chronic comorbidities, previous hospitalizations), and year and hospital fixed effects. 15.3 percent of hospital stays were followed by an unplanned readmission within 30 days. The prevalence of specific social risks ranged from 1.2% (food insecurity) to 13.9% (financial need). Social risk factors are important predictors of unplanned hospital readmission among Veterans after adjusting for medical risk. The risk categories with the strongest adjusted association with 30-day readmission were legal need, risk difference .033 (p=.015); interpersonal violence (r.d.=.022, p<.001); mental health (r.d.=.022, p=.002); social isolation (r.d.=.010, p<.001); and nonspecific psychosocial (r.d.=.017, p<.001). These social risk indicators could be used to target care-transition intervention and follow-up by a social worker to address social needs and avert unplanned hospital readmission.

